# Mendelian randomization analysis of C-reactive protein on septic arthritis risk

**DOI:** 10.1097/MD.0000000000043377

**Published:** 2025-07-18

**Authors:** Hai Lei, Peng Tian, Jun Chen, Shuai Chen, Guochao Lai, Linli Zhou

**Affiliations:** a Department of Orthopedics, The People’s Hospital of Jianyang City, Jianyang, China.

**Keywords:** causal relationship, C-reactive protein, Mendelian randomization, septic arthritis

## Abstract

Several observational studies have shown that C-reactive protein (CRP) is an effective biomarker for evaluating septic arthritis (SA). However, the inherent causal relationship of this connection has not yet been clarified. Thus, this study examined the potential causal association between CRP and SA risk using 2-sample Mendelian randomization (MR). The analysis used 2-sample MR, with the exposure and outcome data from the Genome-Wide Association Study Catalog and FinnGen, respectively. Throughout this study, inverse variance weighting has been used as the primary method. To assess the robustness of the research findings, Cochran *Q* test, Egger regression, and MR pleiotropy residual sum and outlier global tests were conducted. Genetically predicted CRP has a positive causal relationship with the risk of SA (OR_inverse variance weighting_ = 1.37, 95% CI 1.16–1.63, *P* < .001), (OR_MR Egger_ = 1.62, 95% CI 1.20–2.19, *P* = .002), (OR_weighted media_ = 1.48, 95% CI 1.11–1.97, *P* = .008), and (OR_MR PRESSO_ = 1.37, 95% CI 1.18–1.59, *P* < .001). A sensitivity analysis confirmed the robustness of this result. Our study has, for the first time, established a definite causal relationship between CRP and SA risk; that is, an increase in CRP levels leads to an increase in SA risk.

## 1. Introduction

Septic arthritis (SA) is a serious orthopedic disease that can lead to irreversible joint damage and even death. At present, the incidence rate of SA is 6 to 10 cases per 10,000 people,^[1]^ and this data is growing. The previous view was that the cause of SA was the colonization of pathogenic bacteria in the joint cavity, causing infection and subsequently joint injury. There are also views that diseases such as old age, diabetes, cirrhosis, renal dysfunction, and rheumatoid arthritis may be related to the risk of SA.^[2–4]^

Subsequent research revealed that inflammation is pivotal to both the progression and the development of SA.^[5]^ A number of factors are believed to contribute to SA’s development, including environmental influences and genetic factors that result in inflammation.^[6,7]^ C-reactive protein (CRP), as one of the inflammatory markers, is widely recognized as markers of acute inflammation. In previous observational studies, elevated levels of CRP were linked to cardiovascular disease, psoriatic arthritis, type 2 diabetes, idiopathic pulmonary fibrosis, and cancer.^[8–10]^ Several observational studies have identified CRP as a valuable marker for evaluating SA. Studies have shown that erythrocyte sedimentation rate (ESR) and CRP levels are highly sensitive for diagnosing SA with low cutoffs in a 2011 study: 98% for ESR ≥ 10 mm/h, 94% for ESR ≥ 15 mm/h, and 92% for CRP ≥ 2.0 mg/dL (20 mg/L), aiding in the exclusion of SA.^[11]^ According to a 2023 study, a new diagnostic method combining CRP levels (>20 mg/L) and ultrasound effusion evidence (>7 mm) can accurately diagnose SA at 97 percent specificity and 71% sensitivity.^[12]^ Despite these findings, the correlation between CRP levels and SA risk has not been confirmed by genetic studies.^[5,13]^ At the same time, to the best of our knowledge, the intrinsic causal relationship between CRP levels and SA risk has not been reported.

To explore the causal associations between risk factors and diseases, Mendelian randomization (MR), which uses genetic variants as instrumental variables (IVs), has been widely used.^[14]^ Unlike observational studies, this method accounts for biases from confounding factors or reverse causation.^[15]^ The employment of the MR method has unveiled new perspectives on the impact of CRP on SA. And this approach has illuminated the causal connections between CRP and an array of diseases. Notably, Xie et al (2024) leveraged the MR method to prove the causal relationship between CRP and cognitive impairment.^[16]^ And Shi et al (2024) identified a genetic and causal correlation between circulating CRP levels and the risk of lung cancer by using MR methods.^[17]^ Furthermore, Qin et al (2023) explored the causal relationship between CRP and IgA vasculitis through MR method.^[18]^ These pivotal findings underscore the potential of the MR method to offer invaluable insights into devising prevention and treatment strategies for the diseases related to CRP. In pursuit of understanding the connection between CRP-related single-nucleotide polymorphisms (SNPs) and the risk of SA, we embarked on a 2-sample MR analysis. Our study’s robustness was evaluated using a variety of methods, leveraging publicly accessible genome-wide association study (GWAS) summary statistics from OPEN GWAS and FinnGen (R10), with a specific focus on individuals of European descent.

## 2. Method

### 2.1. Study design

**Figure 1.**
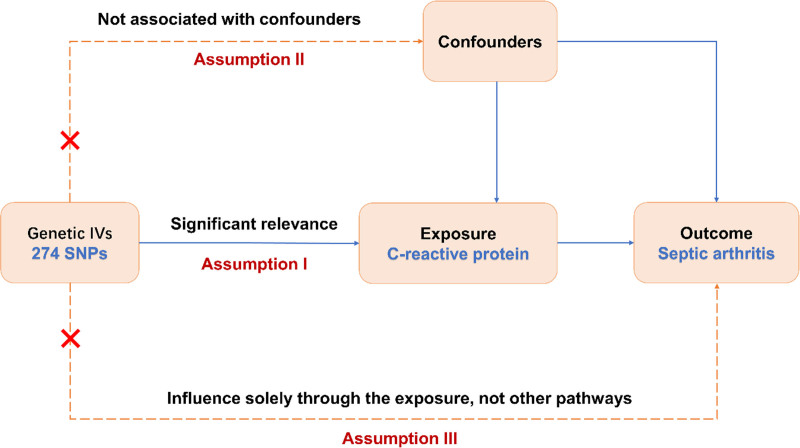
A flowchart of the Mendelian randomization study with the 3 assumptions. SNPs = single-nucleotide polymorphisms.

Our MR analysis, leveraging GWAS summary data, investigated the causal dynamics between CRP levels and the incidence of SA through the examination of 2 distinct cohorts. The IV approach simulates the conditions of a randomized controlled trial by allocating SNPs to offspring in a randomized manner, thereby reducing the impact of confounders such as age and sex. This dual-cohort MR framework is illustrated in Figure 1, showcasing its structure and procedural flow. The genetic instruments chosen for this inquiry are founded on 3 pivotal criteria: Relevance: the genetic instruments must exhibit a significant association with the exposure variable; Independence: the genetic instruments are free from confounding variables, both prior to and following exposure; Exclusion-Restriction: the influence of the genetic instruments on the outcome is strictly mediated through the exposure, excluding any alternative pathways (Fig. 1). This study followed the STROBE-MR guidelines.

### 2.2. GWAS summary data for CRP

CRP GWAS summary data were obtained from a study by British scientists (Said S et al, 2022)^[19]^ that was the largest GWAS on CRP in the Cohorts for Heart and Aging Research in Genomic Epidemiology consortium (N = 575531), and UK Biobank participants (N = 427367). The participants in this project are all of European ancestry, excluding patients with autoimmune diseases and those taking immunomodulatory drugs. Their CRP levels were measured by immunoturbidimetry, and transformed using natural logs. Among the 266 independent loci identified, 211 were reported for the first time. 42 gene sets related to CRP levels were emphasized in gene set analysis (*P* ≤ 3.2 × 10^−6^). Based on organizational expression analysis, CRP-related genes have an undeniable association with liver gene expression and whole blood gene expression.^[19]^ As a result of the phenotype group association study, 27 clinical outcomes were identified as being related to genetically determined CRP. This data was sourced from the GWAS Catalog (https://www.ebi.ac.uk/gwas/home), which does not require additional ethical review and approval.

### 2.3. IVs selection

Implementing rigorous quality control protocols for IV selection consistent with the foundational principles of MR analysis, we ensured the robustness and dependability of our MR study. Initially, genetic markers associated with the exposure were identified, meeting a criterion of genome-wide significance (*P* < 5 × 10^−8^). The subsequent phase involved the exclusion of any SNP significantly correlated with the outcome. In the third stage, SNP clumping was performed with a threshold of 0.001 and a 10 mb window, utilizing the European 1000 genomes project as a benchmark. Lastly, to address potential pleiotropy, the radial regression technique was applied to pinpoint SNPs exhibiting pleiotropic characteristics. Decide whether the IV was invalid Fifth, the F statistic $F=\frac{\beta^{2}}{se^{2}}$ was calculated to, with an F statistic <10 indicating an invalid IV.^[20]^ The accuracy of ambiguous and palindromic SNPs was improved by harmonizing them,^[21]^ and further refinement of SNP selection was achieved with Steiger filtering.^[22]^

### 2.4. GWAS summary data for SA

Data from the FinnGen consortium was used for the SA GWAS summary (R10 version).^[23]^ The FinnGen study represents a collaborative endeavor between the public and private sectors, aiming to advance genomics and personalized healthcare.^[23]^ This initiative has engaged a broad spectrum of participants, including academic institutions, healthcare facilities, the National Institute for Health and Welfare, blood service organizations, biobanks, the Finnish Biobank Cooperative, and multinational pharmaceutical corporations. It has successfully aggregated and examined genomic and health-related information from half a million biobank contributors in Finland, with the objective of elucidating the genetic underpinnings of various diseases. The analysis leverages data from the most recent FinnGen release at the time of submission (R10 version). Within our dataset, designated by the phencode M13_PYOGARTH, there are 2207 cases and 262,844 controls. This dataset defines SA as inflammation in 1 or more joints caused by a bacterial infection within the joint space. Symptoms include pain, stiffness, and decreased range of motion.

### 2.5. Data availability

According to the GWAS Catalog, summary data for CRP is accessible and originates from research conducted by a British academic, as detailed in a publication by Said S et al, in 2022.^[19]^ The study received ethical clearance and full endorsement (11/NW/0382) from the UK Biobank’s ethical review board, adhering to all pertinent ethical standards and regulations. It has been determined that no additional ethical oversight or consent is necessary for this research. The dataset for SA is accessible via the FinnGen website at https://www.finngen.fi/en. All specimens used in this investigation were procured through lawful channels from corresponding biobanks, with each sample receiving approval at the point of acquisition. Given that these datasets were sourced from authorized public repositories, there’s no necessity for further ethical assessment and endorsement.

### 2.6. Statistical analyses

In this study, we used an inverse variance weighting (IVW) analysis as the primary analytical method to investigate the causal relationship between CRP and SA using a 2-sample MR approach. The analysis was conducted using a fixed-effects inverse-variance model.^[24,25]^ In addition, we incorporated complementary methods, including MR-Egger,^[15]^ weighted median,^[26]^ and MR pleiotropy residual sum and outlier (MR-PRESSO).^[27]^ Our next step was to conduct a multistep sensitivity analysis and evaluate both the second and third assumptions. Initially, we used the Cochran *Q* test^[25]^ to determine whether the IVs were heterogeneous. Our next step was to assess the potential horizontal pleiotropy of the IVs by employing MR-Egger regression^[15]^ and MR-PRESSO global test.^[27]^ MR-PRESSO was chosen for its ability to detect and correct for horizontal pleiotropy through outlier removal, which complements MR-Egger focus on testing directional pleiotropy. Further, a leave-one-out analysis was performed to determine whether MR estimates could be affected significantly by a single SNP with a significant horizontal pleiotropic effect. An MR analysis was carried out using the R software (R Foundation for Statistical Computing, Vienna, Austria) environment, utilizing the packages TwoSampleMR, RadialMR, and MRPRESSO.

## 3. Results

### 3.1. IVs for CRP

We utilized 274 SNPs as IVs for CRP. A comprehensive series of quality control procedures, detailed in the Methods section, were applied to assess and validate these SNPs. Notably, each SNP demonstrated sufficient statistical power. The F-statistics for all IVs surpassed 30.08, well above the threshold of *F* > 10. This significant margin indicates that there was no evidence of bias. Detailed information on these SNPs and the outcomes of their quality control evaluations can be found in Table S1, Supplemental Digital Content, https://links.lww.com/MD/P492.

### 3.2. Causal relationship between CRP and SA

**Figure 2.**
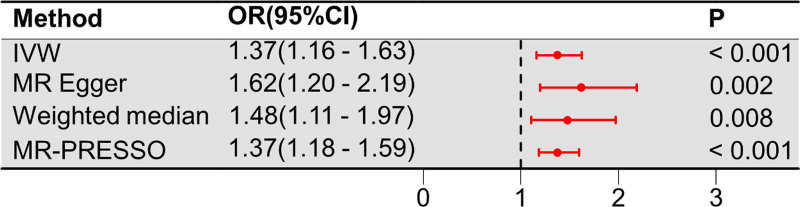
Based on the analysis of the data, CRP is associated with an increased risk of SA. CRP = C-reactive protein, SA = septic arthritis.

**Figure 3.**
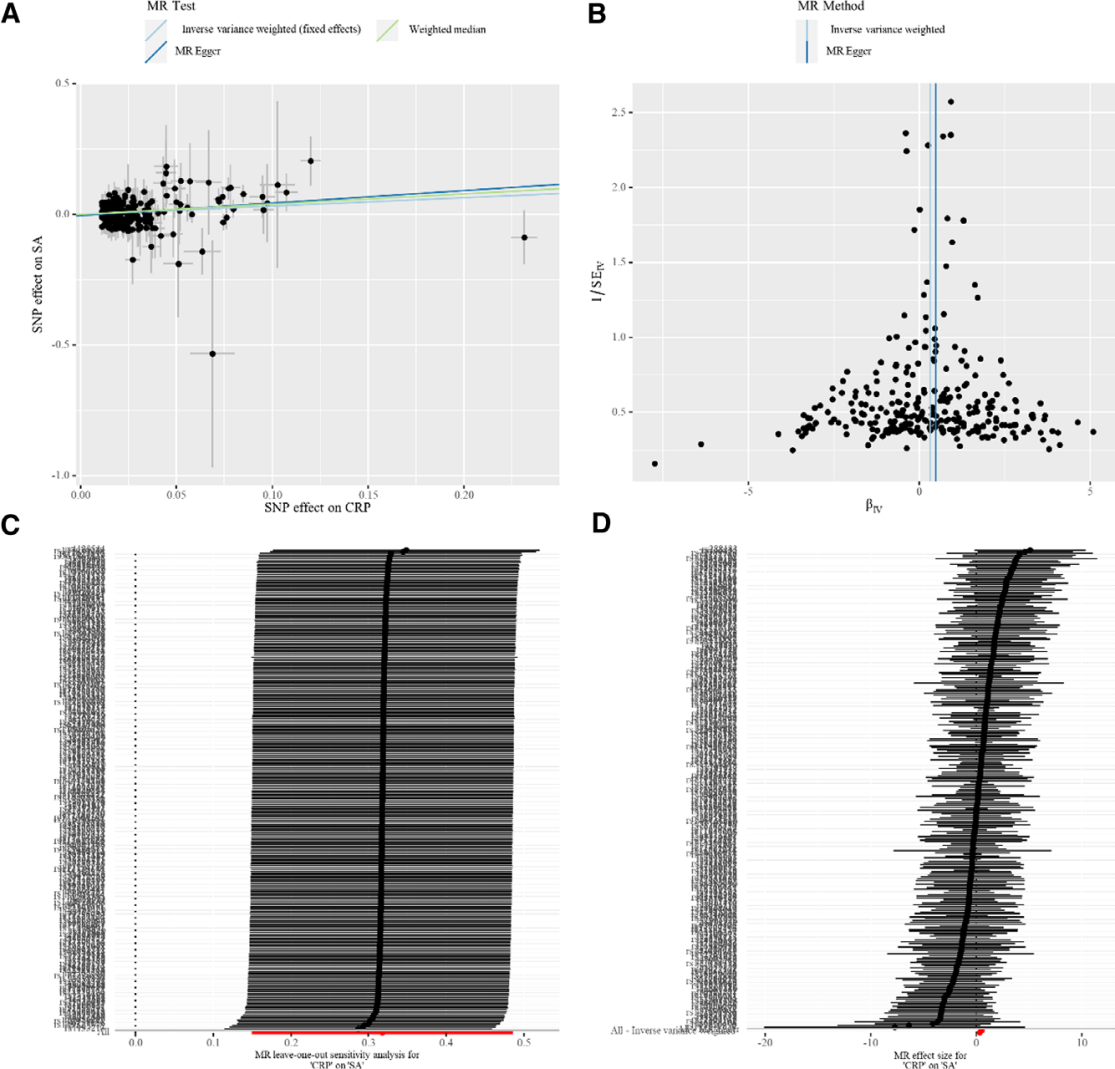
(A) Scatter plots, (B) funnel plots, (C) forests plots, and (D) leave-one-out analyses for C-reactive protein (CRP) in septic arthritis (SA).

The MR analysis results shows that a higher CRP was associated with a higher risk of SA (OR_IVW_ = 1.37, 95% CI 1.16–1.63; *P* < .001), (OR_MR Egger_ = 1.62, 95% CI 1.20–2.19; *P* = .002), (OR_weighted median_ = 1.48, 95% CI 1.11–1.97; *P* = .008), and (OR_MR PRESSO_ = 1.37, 95% CI 1.18–1.59; *P* < .001) according to our analysis (Fig. 2). And the analysis of MR estimates on both CRP and SA is shown in the scatter plot (Fig. 3A). Moreover, the forest plot shows the causal impact of each SNP on SA (Fig. 3C).

### 3.3. Sensitivity analysis

In order to assess the robustness of our results, sensitivity analyses were performed. Initial Cochran *Q* test results indicated that there was no heterogeneity among the IVs (*P*_IVW_ = .997, *P*_MR Egger_ = .997, Table 1), as corroborated by the funnel plot (Fig. 3B). As well, the MR-PRESSO global test (*P* = .997, Table 1) and the MR-Egger regression (*P* = .198, Table 1) did not show any evidence of horizontal pleiotropy among the IVs. This suggests that the IVs are unlikely to influence SA risk through pathways other than those associated with CRP levels. Additionally, using the leave-one-out sensitivity analysis, 1 SNP was removed at a time, stable results were obtained, too (Fig. 3D).

**Table 1:** The result of sensitivity analysis.

Test	Method	Effect size	*P* value
Heterogeneity	*Q* test (MR-Egger)	211.4906	.997
	*Q* test (IVW)	213.1526	.997
Pleiotropy	Egger_intercept	-0.00521	.198
	Global test	215.3824	.997

IVW = inverse variance weighting, MR = Mendelian randomization.

## 4. Discussion

As a serious orthopedic disease, SA rates are on the rise. Many views attribute this phenomenon to an increase in orthopedic infections, an aging population, more invasive procedures, and the growing use of immunosuppressive medicine,^[28]^ while some argue that the risk of SA is related to many intrinsic factors, including old age, diabetes, and chronic diseases.^[1]^ However, there have been numerical observational studies that have highlighted CRP as an independent biomarker for SA and as a systematic marker of inflammation,^[12]^ that is, high levels of CRP may signal persistent inflammation, suggesting an elevated risk of SA. However, these observational studies cannot determine whether there is a certain causal relationship between CRP levels and the risk of SA disease. Through reading published research results, we found that MR methods are widely used to explore the causal relationship between CRP and various diseases.^[16–18]^ Therefore, we chose the MR method to explore this issue. Employing a 2-sample MR method, our study explored the causal impacts of genetic variations linked to serum CRP concentrations on the incidence of SA. This research delineates, for the initial instance, a causal correlation between heightened CRP levels and a heightened susceptibility to SA. And the solidity of these findings was additionally corroborated through various sensitivity analyses. Our findings establish CRP as a function biomarker with both prognostic and therapeutic relevance in SA management. Clinically, CRP quantification demonstrates strong potential for multiple aspects, such as preoperative risk stratification in orthopedic interventions, dynamic monitoring of treatment response through serial CRP velocity measurements, and prognostic triage using CRP-based machine learning algorithms.

The findings of our study support most previous observational studies showing that CRP is positively associated with the risk of SA. George et al observed 70 patients who were admitted with a diagnosis of SA, and found that CRP levels were consistently above 75. The mean number of white blood cells (WBCs) per cubic mm did not reliably indicate infections, while the total WBC number was 13,561/cu.mm, which means that CRP may be more sensitive to SA development than WBC.^[29]^ A retrospective study conducted by Kang et al found that the mean CRP in the SA group was significantly higher than that of the non-SA group for patients with isolated wrist inflammation (mean difference 132 mg/L, 95%CI: 30.9–234).^[30]^ Furthermore, there is evidence that CRP is an important biomarker in the management of SA, and CRP levels may contribute to its pathogenesis and development. A study conducted in 2022 showed that CRP levels were associated with secondary surgery in SA: It is more likely for patients with SA of the native knee joint to undergo a subsequent operation if their initial total white cell count is over 20 109/L after the initial surgery and CRP level falls below 20 % within 24 hours after the initial operation.^[31]^ Meanwhile, it is interesting that although most studies have found that elevated CRP is associated with the occurrence of SA, there are also reports to the contrary. A study by Spyridakis et al examined the clinical characteristics and outcomes of children with culture-negative SA and found these children had lower CRP levels and ESR levels at admission.^[32]^ Our study employs a genetic approach, distinct from traditional observational studies, to circumvent confounding factors, thereby arriving at conclusions that are not only more reliable but also consistent with the majority of existing research. Specifically, our study provides robust evidence supporting a direct genetic causal relationship between elevated CRP levels and SA. Unlike observational studies, which can be susceptible to reverse causality and various confounders, our investigation sidesteps these issues through the application of the MR method.

There is currently no consensus in the academic community on the underlying mechanism of high circulating CRP levels leading to SA risk. Many scholars have provided different studies and speculations, among which the most favored viewpoint is a theory about cytokines. Research has shown that bacterial infections, notably by *Staphylococcus aureus*, are significant risk factors for SA.^[33]^ Genetically manipulated animal models allow for the exploration of host responses to *S. aureus* infections. When macrophage-derived cytokines are removed from the genome, such as lymphotoxin, TNF, and IL-1 receptors, host defenses against *S. aureus* sepsis are reduced, which increases mortality and morbidity rates.^[34]^ When macrophage-derived cytokines are removed from the genome, such as lymphotoxin, TNF, and IL-1 receptors, host defenses against *S. aureus* sepsis are reduced, which increases mortality and morbidity rates.^[35]^ A number of studies have underscored the crucial role inflammatory cytokines play in SA development. And research has demonstrated that the production of CRP is highly dependent on proinflammatory cytokines, particularly IL-6, and to a lesser extent, IL-1 and TNF-α.^[35]^ In addition to stimulating endothelial cells to produce monocyte chemoattractant protein-1, CRP may also directly attract monocytes, suggesting that it may play a role in the development of SA.^[36]^ Additionally, bacterial lipopolysaccharide and IL-4 induce human monocyte chemoattractant protein-1, which binds to 2 receptors, and interferon-gamma.^[37]^ Hence, high levels of circulating CRP may directly or indirectly contribute to SA risk by influencing inflammatory cytokines. These studies provide a possible explanation for how elevated levels of circulating CRP may contribute to the development of SA.^[38]^ In future research, to delineate CRP’s mechanistic role in SA pathogenesis, we recommend a multimodal experimental approach: ex vivo simulation of synovial CRP expression dynamics using *S. aureus*-challenged macrophage-synoviocyte coculture systems; longitudinal tracking of joint destruction patterns in CRP-humanized murine models with intra-articular pathogen inoculation; systems-level integration of CRP-mediated proinflammatory pathways through single-cell RNA sequencing of infected synovial tissue microenvironments.

We offer several strengths in our study, including the first report of analysis of CRP levels in the context of 2-sample MR, about the causal relationship between SA risk and CRP levels. Our study’s results are attributable to several factors. First, 2 large GWAS datasets from European populations were used in this MR study, providing sufficient power to estimate the causal relationship between the 2 variables. After that, we employed an optimal study design incorporating robust MR and sensitivity analyses. We applied a reliable approach to identify potential outliers, enhancing the reliability of our results. Our analysis of heterogeneity among the IVs was based on Cochran *Q* test. We then employed MR-Egger regression and MR-PRESSO global tests in order to assess potential horizontal pleiotropy.^[27]^ Ultimately, our MR analysis reveals that genetically determined CRP levels have a long-term effect on SA risk, minimally influenced by confounders. There is a strong correlation between all IVs and the exposure, as indicated by the total F-statistics value exceeding 10. As a result of the stringent inclusion criteria, our study was not subject to instrument bias due to weak instrumentation.^[15]^ As a consequence, the aforementioned methodologies were used to ensure the reliability and trustworthiness of our results.

The research presented has several constraints that warrant mention. To begin with, the cohort analyzed was exclusively composed of individuals with European descent, casting doubt on the generalizability of these findings to populations of non-European heritage. Different ancestral groups may show disparities in CRP levels and SA risk. And we recommend future MR studies in diverse ancestries to validate our findings. Moreover, while horizontal pleiotropy was not detected, the potential for residual confounding remains, attributed to the unidentified roles of numerous SNPs under investigation. The study’s reliance on aggregated data summaries, owing to the unavailability of participant-specific data, might have veiled biases, impeding the ability to account for variables such as age and sex. Additionally, the analysis focused on CRP levels indicative of chronic exposure effects on SA risk, rather than acute inflammatory reactions. The investigation also overlooked the examination of how variations in CRP levels might modify known confounders or the influence of secondary pathways on SA risk. Despite these shortcomings, the thorough and rigorous approach employed in this study provides compelling evidence of a tentative link between CRP concentrations and the risk of SA, laying the groundwork for subsequent targeted intervention studies and therapeutic explorations.

## 5. Conclusion

In summary, the findings from our investigation imply that an increase in CRP levels circulating in the bloodstream may amplify the likelihood of SA onset. This research may provide fresh perspectives on the exploration of underlying mechanisms contributing to SA’s occurrence and development. Nevertheless, to deepen our understanding of the intricate linkage between CRP levels and SA risk, further studies encompassing biochemistry and intrinsic mechanism domains are essential.

## Author contributions

**Conceptualization:** Guochao Lai.

**Data curation:** Hai Lei.

**Methodology:** Linli Zhou.

**Project administration:** Linli Zhou.

**Writing – original draft:** Hai Lei, Peng Tian, Jun Chen.

**Writing – review & editing:** Shuai Chen, Guochao Lai.

## Supplementary Material


